# Integrated Computational Analysis Highlights unique miRNA Signatures in the Subventricular Zone and Striatum of GM2 Gangliosidosis Animal Models

**DOI:** 10.3390/ijms20133179

**Published:** 2019-06-28

**Authors:** Francesco Morena, Vasileios Oikonomou, Chiara Argentati, Martina Bazzucchi, Carla Emiliani, Angela Gritti, Sabata Martino

**Affiliations:** 1 Department of Chemistry, Biology and Biotechnology, Biochemical and Biotechnological Sciences, University of Perugia, Perugia 06126, Italy; 2 San Raffaele Telethon Institute for Gene Therapy, IRCCS San Raffaele Scientific Institute, Milan 20132, Italy

**Keywords:** Hexosaminidase, lysosomal storage disorders, epigenetics, subventricular zone, striatum

## Abstract

This work explores for the first time the potential contribution of microRNAs (miRNAs) to the pathophysiology of the GM2 gangliosidosis, a group of Lysosomal Storage Diseases. In spite of the genetic origin of GM2 gangliosidosis, the cascade of events leading from the gene/protein defects to the cell dysfunction and death is not fully elucidated. At present, there is no cure for patients. Taking advantage of the animal models of two forms of GM2 gangliosidosis, Tay-Sachs (TSD) and Sandhoff (SD) diseases, we performed a microRNA screening in the brain subventricular zone (SVZ) and striatum (STR), which feature the neurogenesis and neurodegeneration states, respectively, in adult mutant mice. We found abnormal expression of a panel of miRNAs involved in lipid metabolism, CNS development and homeostasis, and neuropathological processes, highlighting region- and disease-specific profiles of miRNA expression. Moreover, by using a computational analysis approach, we identified a unique disease- (SD or TSD) and brain region-specific (SVZ vs. STR) miRNAs signatures of predicted networks potentially related to the pathogenesis of the diseases. These results may contribute to the understanding of GM2 gangliosidosis pathophysiology, with the aim of developing effective treatments.

## 1. Introduction

The GM2-Gangliosidosis, Tay-Sachs (TSD; OMIM Entry #272800) and Sandhoff (SD; OMIM Entry #268800) diseases are autosomal recessive fatal neurodegenerative disorders caused by the deficiency of the lysosomal enzyme β-Hexosaminidase (Hex, E.C.3.2.1.52). The latter exists in two major isoenzymes: the β-Hexosaminidase A (HexA), composed of α-subunit (HEXA gene; chromosome 15) and β-subunit (HEXB gene; chromosome 5), and the β-Hexosaminidase B (HexB) composed of β-subunits [1,2]. Mutations in the HEXA gene cause the absence of HexA and lead to TSD [1,2]. Mutations in the HEXB cause the absence of both HexA and HexB and lead to SD [1,2], where, as a consequence, arises the β-Hexosaminidase S, a minor Hex isoenzyme consisting of α-subunit and lacking of activity in vivo [3,4]. Both HexA and HexB hydrolyze *N*-acetylgalactosamine/*N*-acetylglucosamine residues from glycoproteins, glycolipids and glycosaminoglycans, but only HexA is capable to hydrolyze the GM2 ganglioside [5,6]. Thus, the absence of HexA and/or HexB leads to the storage of undegraded substrates in the lysosomes, culminating with cell dysfunction and death [1]. The main hallmark of both TSD and SD is the accumulation of undegraded GM2 ganglioside and related lipids within the nervous system [7,8], leading to progressive neurodegeneration that ends with death in the second or third year of the life of affected children [8]. No cure is currently available, although many groups are testing several experimental approaches in animal models [1,9,10,11,12,13]. The main challenges hampering the development of successful treatments are mostly related to the complex and yet not fully elucidated pathogenesis of the diseases, due to the general inaccessibility of human tissues, in particular at the first stage of the disease. At present, much of our understanding of GM2 gangliosidosis derives from studies focused on the analysis of the correlation between Hex deficiency, lipid storage, and neurodegeneration/ neuroinflammation [9,10,11,12,13,14] in the SD and TSD animal models [14,15,16].

Recently, microRNAs (miRNAs) are emerging as key contributors to the etiology of diseases and to the clinical aberration observed even in monogenic disorders [17,18,19,20]. miRNAs are a class of non-coding RNAs that regulate gene expression in animals and plants at post-transcriptional level [21,22]. In fact, by the microRNA Responsive Element, miRNAs bind messenger RNA (mRNA) targets to seed sequences, and activate their degradation/translational inhibition [21,22]. The ratio of miRNA-mRNA is heavily unbalanced as one miRNA may regulate hundreds of mRNAs [22,23,24,25]. Moreover, it has been demonstrated that the activity of miRNAs is controlled by the concentration of their targets and that the latter may influence the function of a miRNA toward its other targets [26,27]. The role of miRNA has been documented in several physiological and pathological conditions [17,27]. miRNAs regulate cell cycle [28], cellular metabolism [29], epigenetic mechanisms [30], immunity mechanisms [31], stem cell differentiation [32] and cell reprogramming [33]. Of note, abnormal expression of miRNAs should affect the same biological processes that they have regulated and, as a consequence, lead to numerous diseases [17,18,34,35]. miRNA-mediated regulation of gene expression plays a key role in fate determination of neuronal cells, neuronal circuit development, maturation and function [36,37]. Also, miRNAs are often dysregulated in neurodegenerative disorders [38,39,40,41,42,43,44,45]. Currently, there are few studies exploring the involvement of miRNAs in Lysosomal Storage Diseases (LSDs) [20,46,47], while the potential impact of dysregulated miRNA expression in the pathogenesis of GM2 gangliosidosis has not been studied.

In this work, we investigated the contribution of miRNAs in the GM2 gangliosidosis. We performed an integrated computational analysis of a panel of 12 miRNAs selected based on their predicted involvement in lipid metabolism [48,49], regulation of CNS development and homeostasis [48,49], and neuropathological processes (i.e., neurodegeneration, inflammation) that characterize GM2 gangliosidosis.

We performed the study in two brain regions, the subventricular zone (SVZ) and the striatum (STR), that, respectively, represented the neurogenesis and neurodegeneration states of adult murine TSD and SD animal models [15,16,50]. The SVZ and STR have anatomical contiguity [51,52] and functional connections [53,54,55], but they have a distinctive cellular organization. The SVZ is the major stem cell niche in the mammalian post-natal/adult brain [56,57] and comprises neural stem cells endowed with intrinsic biological features that distinguish them from lineage-committed progenitors and mature brain cells [58]. The STR is part of the basal ganglia of the forebrain [51,59,60]. It comprises GABAergic neurons, different classes of GABAergic and Cholinergic interneurons that manage multiple features of cognition (e.g., decision-making, reinforcement, motivation, both motor and action planning, and reward perception [59,60]). Of note, the STR is highly neurodegenerated in GM2 gangliosidosis and, as a consequence, almost all of the abovementioned neurologic functions are impaired [9,61,62].

We found an altered expression of some of the miRNAs investigated in SD vs. WT and TSD vs. WT, and identified a unique disease- (SD or TSD) and brain region-specific (SVZ vs. STR) profile of miRNA expression. We also showed the correlation of predicted microRNAs pathways with molecular events described in SD and TSD. The overall findings of this study may contribute to the understanding of pathophysiology of GM2 gangliosidosis.

## 2. Results

### 2.1. Isolation of SVZ and STR tissues from the brain of adult mice

**Figure 1:**
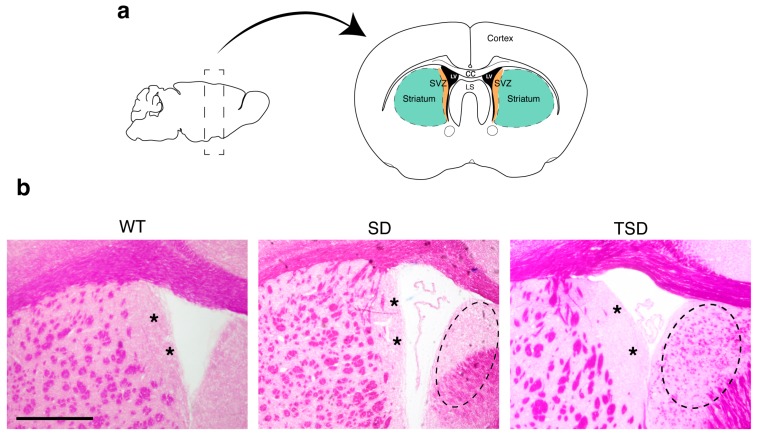
SVZ and STR tissues isolated from WT, TSD and SD mice. (**a**) Schematic of brain dissection: with two cuts we isolate a slice of tissue comprising the lateral ventricles. The periventricular area (SVZ; orange) and a portion of striatum (STR; green) are carefully dissected out from both hemispheres using small forceps. Black, lateral ventricles (LV); white, corpus callosum (cc); lateral septal (LS). (**b**) Representative images of PAS staining on vibratome-cut brain coronal sections from SD, TSD and WT mice. The asterisks indicate the SVZ region. The dotted ellipse indicates representative pathological changes Scale bar: 300 µm.

The SD mice (hexb−/−) recapitulate the severe infantile form of SD (abundant storage of GM2 ganglioside in the nervous system, progressive neurodegeneration starting from 3 months of age and death at 4 months) [9,15,16]. The TSD mice (hexa−/−) recapitulate the mild form of TSD (delayed storage of GM2 ganglioside in the nervous system, slow progressive neurodegeneration, and normal life-span [15,16,50]). The SVZ and the STR were isolated from the brain of SD and TSD mice at the end-stage of the disease (4 and 18 months old mice, SD and TSD, respectively) [16] and from age-matched wild-type (WT) mice according to described protocols [63] (Figure 1a). In Figure 1b are reported representative Periodic acid–Schiff (PAS) staining of vibratome-cut brain coronal sections from TSD, SD, and age-matched WT controls in order to highlight glycolipid storage (one of the main hallmarks of the disease, [9,15,16,62]. Brain coronal section showed that the periventricular region, and specifically the lateral SVZ, were PAS-negative in SD, TSD, and WT controls (Figure 1b, asterisks). Conversely, a more intense and widespread PAS-positive staining was detected in the in Lateral septal nucleus as well as in the STR parenchyma of TSD and SD mice as compared to WT controls, in which PAS+ myelin-rich patches are expected as described by Mengler and co-authors [64] (Figure 1b).

### 2.2. Differential miRNA expression analysis in SVZ and STR of SD and TSD mice

We analyzed a panel of 12 miRNAs (miR-9, miR-9*, miR-19a, miR-29a, miR-33, miR-34a, miR-124, miR-126a, miR-128, miR-133a, miR-137, and miR-181c) selected based on their involvement in controlling: neurogenesis, neurodevelopment, neurodegeneration, inflammation, stem cell properties and lipid metabolism (from literature open access papers; miRBASE [48]; Figure S1 for details).

**Figure 2:**
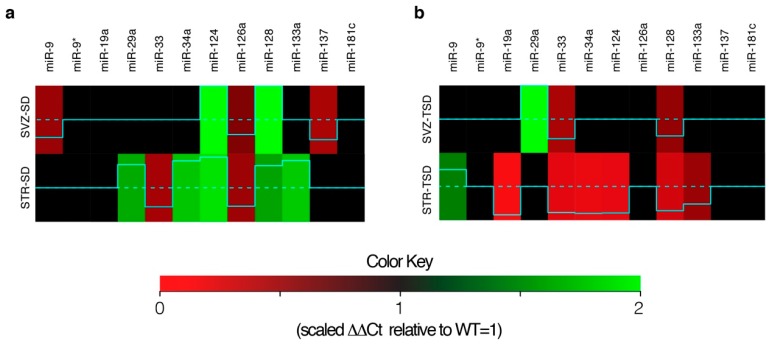
Heat map of miRNAs expression profile. (**a**) SVZ-SD and STR-SD; (**b**) SVZ-TSD and STR-TSD. Color nuance represents relative increase (green)/decrease (red) in miRNAs expression with respect to the related controls, SVZ-WT and STR-WT (blue dashed line). The dark color indicates a comparable expression of miRNAs in GM2 gangliosidosis mice and WT counterparts.

In Figure 2 is reported the differential expression analysis of all miRNAs in SVZ and STR from SD and TSD mice with respect to the WT counterparts (blue dashed line). In SVZ-SD we found 5 miRNAs significantly changed as compared to SVZ-WT, with 2 miRNAs upregulated (miR-124, miR-128) and 3 miRNAs downregulated (miR-9, miR-126a, miR-137) (heat maps in Figure 2a). In STR-SD the expression of 7 miRNAs was significantly different as compared to the STR-WT, with 5 miRNAs upregulated (miR-29a, miR-34a, miR-124, miR-128, miR-133a), and 2 miRNAs downregulated (miR-33, miR-126a) (heat maps in Figure 2a). In SVZ-TSD the expression of 3 miRNAs was significantly varied as compared to the SVZ-WT, with 1 miRNA upregulated (miR-29a), and 2 miRNAs downregulated (miR-33, miR-128) (heat maps in Figure 2b). In STR-TSD the expression of 7 miRNAs was significantly changed as compared to STR-WT, with 1 miRNA upregulated (miR-9) and 6 miRNAs downregulated (miR-19a, miR-33, miR-34a, miR-124, miR-128, miR-133a) (heat maps in Figure 2b).

Next, we applied a filtering approach that compares only significant differentially expressed miRNAs in SVZ-SD vs. SVZ-WT, STR-SD vs. STR-WT, SVZ-TSD vs. SVZ-WT, STR-TSD vs. STR-WT, respectively, adjusted for *p*-value < 0.05 and Log_2_ fold change < 0.5 or >1 (Table 1). Accordingly, in SVZ-SD vs. SVZ-WT and STR-SD vs. STR-WT we identified 4 miRNAs downregulated (miR-9, miR-33, miR-126a, miR-137) and 2 miRNAs upregulated (miR-124, miR-128) (Table 1); whereas in SVZ-TSD vs. SVZ-WT, STR-TSD vs. STR-WT we identified 5 miRNAs downregulated (miR-19a, miR-33, miR-34a, miR-124, miR-128) and 1 miRNAs upregulated (miR-29a) (Table 1).

**Figure 3:**
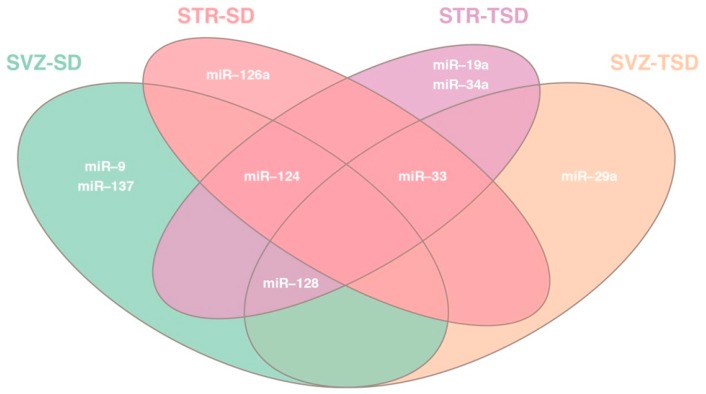
Venn diagram of differentially expressed miRNAs analysis in SVZ and STR of SD and TSD mice. Shown are miRNAs shared between both brain areas in SVZ and STR of both GM2 gangliosidosis mice, and miRNA(s) that is/are characteristic of one brain region only.

From now, all data refer to the panel of miRNAs differentially expressed with statistical significance and Log_2_ fold change (Table 1). Interestingly, some of these miRNAs are shared between the two brain areas in both GM2 gangliosidosis mice, while other are characteristics of one region (Venn diagram in Figure 3). Thus: (i) miR-124 is shared by SVZ-SD, STR-SD and STR-TSD; (ii) miR-128 is shared by SVZ-SD, STR-TSD and SZV-TSD; (iii) miR-33 is shared by STR-SD, STR-TSD and SVZ-TSD; (iv) miR-9 and miR-137 are exclusive of SVZ-SD; (v) miR-126a is distinctive of STR-SD; (vi) miR-19a and miR-34a are typical of STR-TSD; (vii) miR-29a is proper of SVZ-TSD (Figure 3).

### 2.3. Target prediction and gene list analysis in SVZ and STR of SD and TSD mice

**Figure 4:**
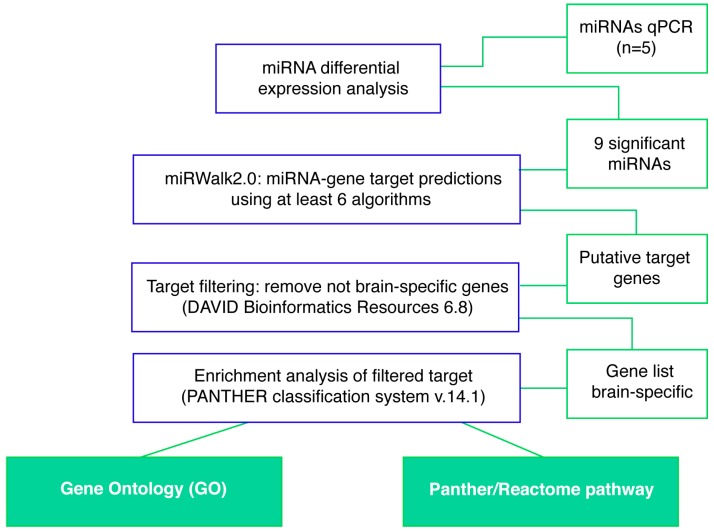
Workflow of the integrative analysis of miRNA-target prediction. The expression data included the dataset derived from miRNA qPCR profiling. Predicted miRNA-target by miRWalk2.0 was filtered for retaining only brain-specific genes and evaluated for gene ontology terms (GO) and specific pathway enrichments (Panther/Reactome).

Next, we performed a miRNA-mRNA target prediction analysis in order to generate a miRNA signature of brain-specific molecular pathways and potentially related to the pathogenesis of GM2 gangliosidosis. To avoid false positive microRNA targets prediction, only targets estimated by at least 6 algorithms plus all experimentally validated targets were taken into account (Figure 4).

The summary of target counts is shown in Table 2. miR-9 and miR-124 were the miRNAs with the highest number of count targets, whereas miR-126a and miR-137 were the miRNAs with the lowest number of total count targets. However, all selected miRNAs guaranteed the prediction of a list of target genes > 200. The predicted genes’ list for each miRNA was then forwarded to DAVID external resources, and filtered for brain tissue-specific gene expression (Figure 4, Table 2).

**Figure 5:**
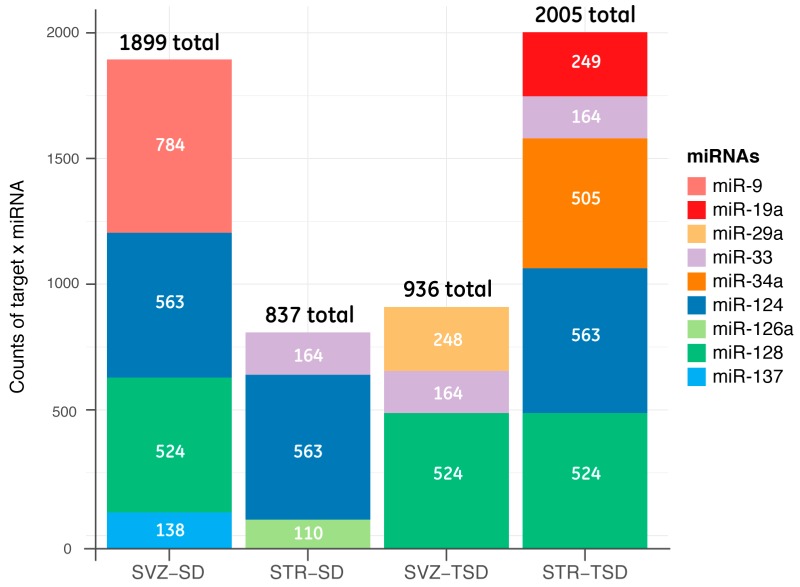
Counts of validated and predicted target genes for each miRNA in SVZ and STR regions of SD and TSD mice. The stacked bar plot indicates the numbers of target genes for each miRNA (color bar) within the brain regions of hex-null mice.

This step allowed us to identify genes with a potential different expression that were correlated with each candidate miRNA in SVZ and STR brain regions. Hence, we identified 1899 total target genes in SVZ-SD regulated by miR-9, miR-124, miR-128 and miR-137; 837 total target genes in STR-SD regulated by miR-33, miR-124 and miR-126a; 936 total target genes in SVZ-TSD regulated by miR-29a, miR-33, miR-128; 2005 total target genes in STR-TSD regulated by miR-19a, miR-33, miR-34a, miR-124 and miR-128 (Figure 5).

### 2.4. The impact of miRNA meta-signature on cellular pathways and biological processes in SVZ and STR of SD and TSD mice

**Figure 6:**
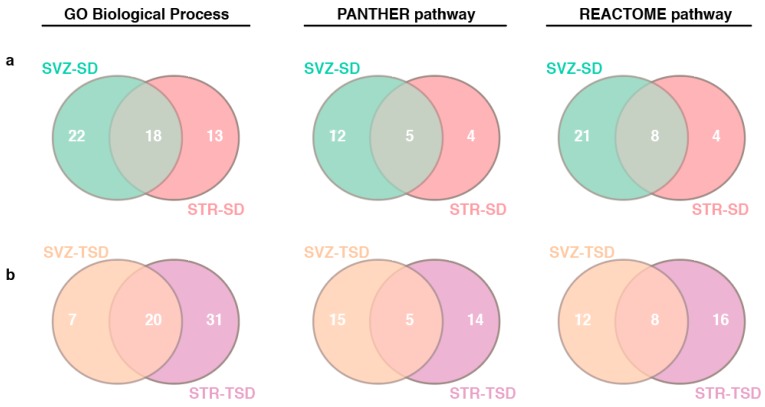
Venn diagram of GO BP and pathways enrichment in SVZ and STR of GM2 gangliosidosis. Shown are overlapping and non-overlapping biological process and pathways between (**a**) SD-SVZ and SD-STR; (**b**) TS-SVZ and TS-STR.

**Figure 7:**
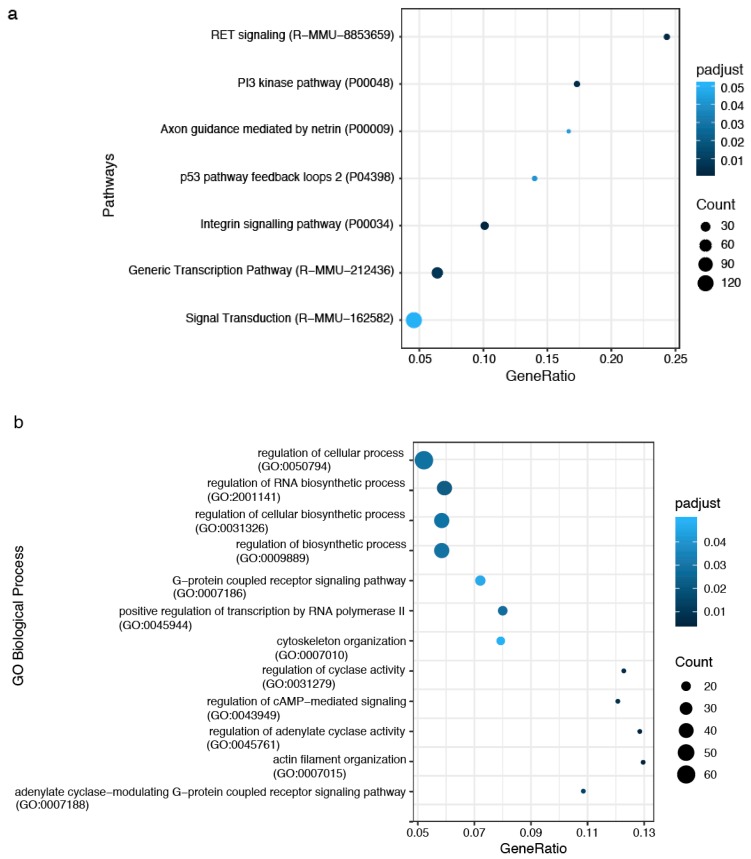
Dotplot of enrichment analysis in SD-STR compared to SD-SVZ. (**a**) Panther (P) and Reactome (R-MMU) pathways; (**b**) Gene Ontology Biological Process. *p*-adjusted < 0.05 was considered statistically significant.

**Figure 8:**
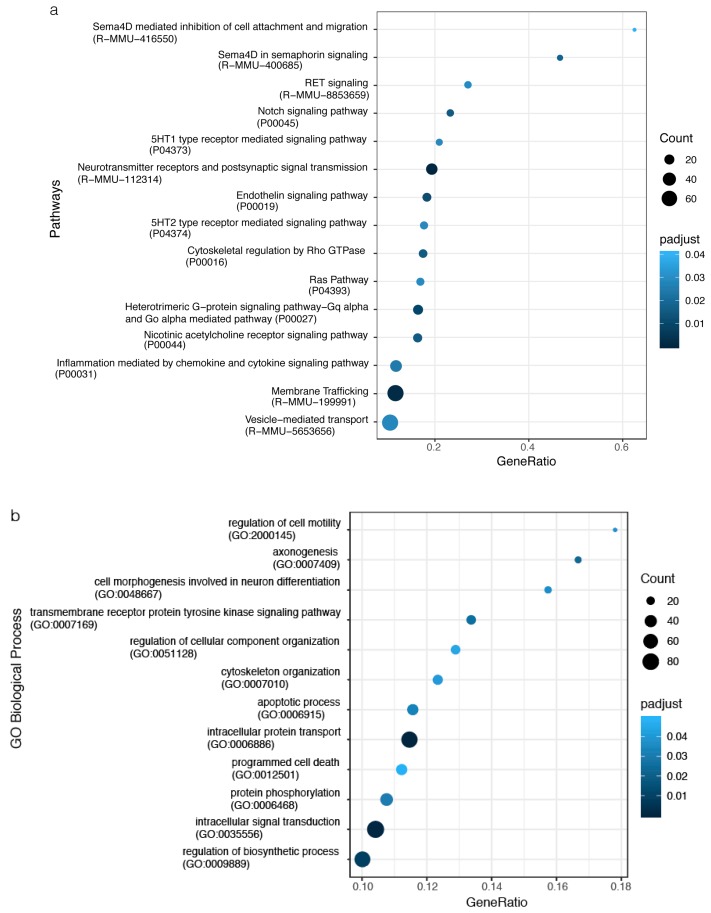
Dotplot of enrichment analysis in TS-STR compared to TS-SVZ. (**a**) Panther (P) and Reactome (R-MMU) pathways; (**b**) Gene Ontology Biological Process. *p*-adjusted < 0.05 was considered statistically significant.

All identified target genes were further analyzed for enrichment analysis by the PANTHERDB v.14.1 classification system web tool [65]. This software allowed acquisition of knowledge about the biological relevance of each miRNA. We obtained signatures of the Gene Ontology Biological Process (GO BP), Panther pathways and Reactome pathways (Figure 6, Figure 7, Figure 8 and Figure S2).

In order to correlate these findings with the pathophysiology of GM2 gangliosidosis, wherever possible, we performed a comparative analysis of the enriched pathways (*p* < 0.05) in the neurodegenerative area (STR) versus the neurogenetic area (SVZ). The comparative analysis allowed us to erase the shared pathways between SVZ and STR in each model and to highlight over-represented STR-specific processes (Figure 6, Venn diagram). We identified 13 GO BP, 4 Panther pathways and 4 Reactome pathways enriched in STR-SD (*p*-adjusted < 0.05) (Figure 6a); 22 GO BP, 12 Panther pathways and 21 Reactome pathways enriched in SVZ-SD (*p*-adjusted < 0.05) (Figure 6a); 31 GO BP, 14 Panther pathways and 16 Reactome pathways enriched (*p*-adjusted < 0.05) in STR-TSD (Figure 6b); 7 GO BP, 15 Panther pathways and 12 Reactome pathways enriched (*p*-adjusted < 0.05) in SVZ-TSD (Figure 6b).

Enriched Panther pathways and Reactome pathways in STR-SD are most frequently associated with cell signaling (Integrin signaling pathway, RET signaling pathway, Signal Transduction pathway), PI3 kinase pathway, axon guidance mediated by netrin, p53 pathways feedback loops 2 and generic transcription pathway (Figure 7a). The enriched GO BP included regulation of adenylate cyclase activity, actin filament organization, cytoskeleton organization and G-protein coupled receptor signaling pathway, cellular processes, RNA biosynthetic process, cellular biosynthetic process, transcription by RNA Polymerase II (Figure 7b).

Enriched Panther pathways and Reactome pathways in STR-TSD envisaged pathways frequently associated with cell signaling (5HT1 and 5HT2 type receptor mediated signaling pathway, Inflammation mediated by chemokine and cytokine signaling pathway, Notch signaling pathway, Ras pathway, Nicotinic acetylcholine receptor signaling pathway, Endothelin signaling pathway, Heterotrimeric G-protein signaling pathway, RET signaling, Sema4d in semaphorin signaling), cytoskeletal regulation by Rho GTPase, membrane trafficking, Sema4d mediated inhibition of cell attachment and migration, vesicle mediated transport and neurotransmitter receptors and postsynaptic signal transmission (Figure 8a). The most enriched GO BP included regulation of biosynthetic process, cytoskeleton organization, cell morphogenesis in neuron differentiation, programmed cell death, intracellular protein transport, apoptotic process, intracellular signaling transduction, axogenesis, regulation of cell motility, protein phosphorylation, transmembrane receptor protein tyrosine kinase signaling pathway and regulation of cellular component organization (Figure 8b).

### 2.5. Identification of miRNA-mRNA target-relationship in enriched pathways

**Figure 9:**
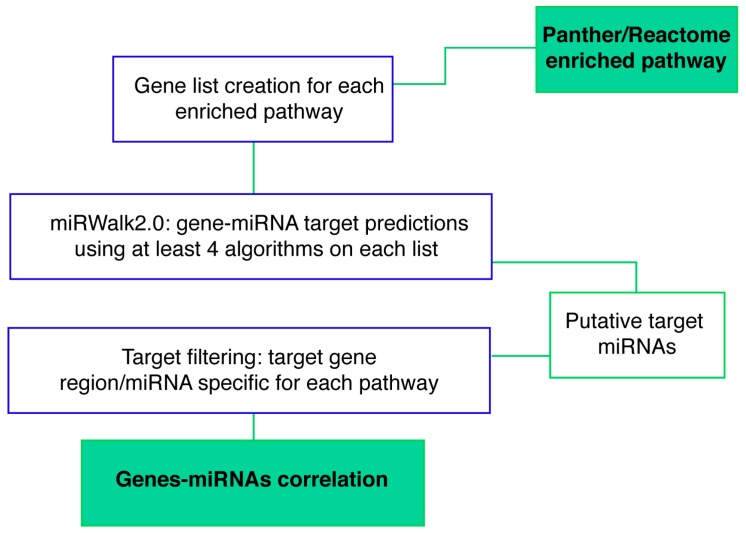
Workflow of the integrative analysis of Gene-miRNA target prediction. The input data included the target genelist of each enriched pathway. Predicted gene-miRNA by miRWalk2.0 was filtered for specific miRNA and specific brain region (SVZ, STR), and gene-miRNA correlation was identified.

Finally, we performed a Gene-miRNA-Network meta-analysis in order to identify the genes of each enriched pathways down-stream of each miRNA (Figure 9). The main enriched pathways and target genes are reported in Table S1 (supplementary file). Of note, some genes were down-stream of several miRNAs while some/other genes were shared among different pathways, confirming the crosstalk activity of miRNAs.

## 3. Discussion

In this work, we explored the contribution of miRNAs in the pathophysiology of the GM2 gangliosidosis, Tay-Sachs and Sandhoff diseases. We identified a panel of 9 miRNAs (miR-9, miR-19a, miR-29a, miR-33, miR-34a, miR-124, miR-126a, miR-128 and, miR-137) with altered expression in the SVZ and the STR of both SD and TSD mice with respect to the age-matched WT counterparts. We documented a peculiar distribution and composition of the abovementioned miRNAs in the SVZ and STR of both GM2 gangliosidosis animal models, with some miRNAs shared by both brain regions and others exclusive of SVZ or STR. Thus, we identified unique miRNAs profiles for SVZ and STR in SD and TSD mice and revealed a STR- or SVZ-specific signature of altered pathways in SD or TSD. After elaboration with computational analysis, we highlighted molecular interplays potentially associated with the neurodegenerative condition in SD and/or TSD mice.

In STR-SD, the miRNA profile was composed of miR-33, miR-124, and miR-126a. The latter was STR-SD-specific. The miRNA cluster has several common predicted downstream pathways that, if altered, may concur to the neurodegenerative process in SD. For instance, miR-33, miR-124, and miR-126a control Netrin3 and the netrin-receptor (Dcc) genes within Axon netrin pathway, which meta-analysis correlated with the abnormal dendritic-like growth processes described in feline GM2 gangliosidosis [66,67], and with the impaired neurite outgrowth in the retina documented in SD mice [68]. Moreover, miR-33, miR-124 and miR-126a regulate the Integrin pathway that include genes involved in the regulation of cell survival, cell proliferation, cell differentiation, cell adhesion, spreading and migration. Within these target genes we highlighted the Rag GTPase, a class of proteins involved in the activation of the recruitment of mTORC1 to the lysosome by aminoacids, a pathway associated with dysfunctions of the lysosomal compartment [69].

In STR-TSD, we identified an miRNA profile composed of miR-19a, miR-33, miR-34a, miR-124, and miR-128. Among these, miR-19a and miR-34a were STR-TSD-specific. Yet, the computational analysis revealed that miR-34a, miR-124, and miR-128, and to a lower extent, miR-33 and miR-19a, govern different genes involved in cytoskeletal organization, neurotransmission, inflammation, membrane trafficking, and vesicle-mediated transport. All these pathways correlated with biological processes that are altered in GM2 gangliosidosis, in particular with target genes of the vesicle-mediated transport pathways. It has been shown that, due to primary defects in lipid catabolism in LSDs, the storage of sphingolipids in the lysosomes hampers the intracellular distribution of cholesterol, leading to the alteration of the lipid membrane composition and, consequently, affecting the trafficking of sphingolipids and proteins [70]. In this regard, in attempt to develop a therapeutic treatment for TSD, we have demonstrated that HexA delivered in the culture medium was efficiently up-taken by TSD fibroblasts, but the enzyme failed to reach the lysosomes in a sufficient quantity to guarantee the hydrolysis of the GM2 ganglioside to GM3 ganglioside. We interpreted these results as a consequence of the lipid catabolic alteration that affected the correct sorting of the recaptured HexA within the cells [71]. Further evidence correlating the alteration of lysosomal enzymes trafficking and miRNAs activities derives from one of the target genes of miR-34a, the Cation-dependent mannose-6-phosphate receptor (M6PR), that represents the main mechanism for endocytosis of the lysosomal enzymes. The M6PR transports manno-6-phosphorylated lysosomal enzymes, including HexA and HexB, from the Golgi Apparatus to the lysosomes or to the plasma membrane and, via receptor-mediated endocytosis, from the cell surface to lysosomes [72,73]. We also highlighted Sortilin, a receptor required for the transport of protein from the Golgi Apparatus to lysosomes via M6PR-independent pathway [74], as a predicted target under the control of miR-128. Other predicted genes down-stream of miR-34a and miR-124 are involved in the networks of neurotransmitter receptors and postsynaptic signal transmission. These miRNAs are potentially involved in the dysfunction of the GABAergic neurons and GABAergic interneurons in the STR-TSD, as shown by the disappearance of the myokymia and improvement in the ataxia after the administration of GABAergic drugs Gabapentin and Tiagabine in one case of adult GM2 gangliosidosis, B1-variant [75]. Finally, we focused on target genes of miR-33 involved in the Ca2+ transport from/into the endoplasmic reticulum such as Calcium/Calmodulin Dependent Protein Kinase II Gamma (CAMK2G) that, in neurons, may partake in the rise of dendritic spines, formation of synapses, and maintenance of synaptic plasticity. There is no direct evidence describing the role of CAMK2G in GM2 gangliosidosis. However, alterations of calcium homeostasis in the endoplasmic reticulum have been described for GM2 gangliosidosis as well as for other LSDs [76]. For instance, it has been demonstrated that GM2 ganglioside inhibits calcium re-uptake into the endoplasmic reticulum by decreasing the activity of the sarco/endoplasmic reticulum Ca^2+^ ATPase (SERCA) [77].

In SVZ-SD, the miRNA profile consisted of miR-9, miR-124, miR-128 and miR-137. Here, miR-9 and miR-137 are exclusive to SVZ-SD. The computational analysis emphasized unique downstream target pathways in SVZ-SD that do not overlap with STR-SD. This envisages genes associated with neurotransmitter receptors and growth factors. Among them, we highlighted a correlation between the Hexosaminidase metabolism and the activity of Epidermal growth factor receptor (EGFR), inasmuch as the ganglioside GM3 is one of its negative modulators [78]. Additionally, it has been shown that EGFR activity is associated with the abnormal accumulation of heparan sulfate in mucopolysaccharidosis IIIB, due to the absence of the lysosomal enzyme α-N-acetylglucosaminidase [79]. Target genes controlled by miR-33 and miR-128 belong to the Insulin-like growth factors (IGF) pathways and are abundant also in the brain. In particular, the IGFII binds to the plasma membrane IGF-II/mannose-6-phosphate receptor taking part in the sorting processes allowing M6P-tagged proteins - such as lysosomal enzymes- to be up-taken from the plasma membrane for lysosomal delivery [80]. The synergy of miR-9, miR-33, miR-124 and miR-137 also control axon guidance genes involved in the Slit Robo pathway, and inflammation-related genes involved in chemokine and cytokine signaling pathway, which is particularly relevant in GM2 gangliosidosis [81]. We also underlined the Sialic acid metabolism pathways, under the activity of miR-128, which is relevant for the synthesis of gangliosides and other glycosphingolipids (the most abundant sialoglycans in neural cells) [82].

In SVZ-TSD, we identified an miRNA profile composed of miR-29a, miR-33, and miR-128, with miR-29a being SVZ-TSD-specific. Yet, the computational analysis highlighted pathways that were not shared with STR-TSD. Axon guidance mediated by netrin is downstream to miR-29a and miR-33, while the Axon guidance mediated by Slit Robo, the Transforming Growth Factor beta signaling (TGF-ß), the Synthesis of Phosphatidylinositol phosphates (PIPs) at the plasma membrane and the Synthesis of PIPs at early endosomes membrane pathways were downstream to miR-33 and miR-128. A recent work has correlated some of the abovementioned pathways with LSDs. It was demonstrated that in a model of mucolipidosis II, the loss of carbohydrate-dependent lysosomal sorting affects the activity of several cathepsin proteases via TGF-β [83]. Other important pathways downstream to miR-29a include the Insulin/IGF pathway, the protein kinase MAP kinase signaling cascade, Phosphoinositide 3-kinases (PI3 kinase) pathway, neurotransmitters pathway and Integrin signaling pathway that, as reported above, might be correlated with biological processes altered in GM2 gangliosidosis.

Interestingly, among the miRNAs investigated, miR-124 and miR-128 were expressed in opposite direction in SD and TSD mice. In fact, miR-124 was upregulated in STR-SD and downregulated in STR-TSD, whereas miR-128 was upregulated in SVZ-SD and downregulated in SVZ-TSD. We speculated that this tailored expression might correlate with the different features and disease progression in SD and TSD mice [15,16].

In conclusion, these results showed altered expression of several miRNAs in the SVZ and STR of SD and TSD mice whose predicted target genes belong to signaling/biological pathways with documented or suggested dysregulation in GM2 gangliosidosis as well as in other LSDs. By exploring GM2 gangliosidosis pathophysiology at the molecular level, we highlighted a novel correlation between the activity of miRNAs, neurogenesis (SVZ) and neurodegeneration/neuroinflammation (STR) in SD and TSD murine models. The robustness of our findings is validated by the characteristics of the murine models used, because WT, SD and TSD mice are inbred animals and each model has identical genotype. The meta-data provided by the computational analysis performed here may be a suitable tool for better elucidating the mechanisms downstream to the primary storage, which may contribute to the progression of GM2 gangliosidosis neuropathology, thus aiding the developing of more proficient therapeutic approaches.

## 4. Materials and Methods

### 4.1. Animal Models

SD (hexb−/−, kindly provided by Platt F. M.) and TSD mice (hexa−/−) were generated as previously described [15,50] and were bred in the SPF animal house at the San Raffaele Scientific Institute. Genotyping was performed by DNA extraction from tail tips according as described previously [84]. C57/bL6 (Charles-River, Calco, LC, Italy) and hexb+/+ littermates were used as WT controls for hexa−/− and hexb−/− mice, respectively. All procedures involving mice were performed according to protocols approved by an internal Animal Care and Use Committee (IACUC #791) and were reported to the Italian Ministry of Health (Authorisation n.923/2016-PR, released on 5 October 2016).

### 4.2. Tissue Dissection

SD (4 months of age; *n* = 5), TSD (18 months of age; *n* = 5), and age-matched control mice (WT, *n* = 5) were sacrificed by overdose of Avertin. Brains were isolated and olfactory bulbs were removed. A coronal slice comprising the periventricular subventricular zone (SVZ) and the striatal region (STR) was cut. The periventricular tissue and the central portion of the STR were then carefully dissected out from both hemispheres of the same tissue slice. Dissected tissues were washed in PBS and immediately frozen in liquid nitrogen. The tissues (SVZ or STR) from the two hemispheres of the same brain were pooled in one tube.

### 4.3. Periodic Acid Shiff (PAS) Staining

Mice were anesthetized intraperitoneally with Avertin and perfused with 4% paraformaldehyde (PFA) in PBS. Brains were removed, equilibrated for 24 h in 30% sucrose in PBS and quickly frozen in optimal cutting temperature compound (OCT). Then, 20-μm-thick serial coronal cryostatic sections were processed for PAS. The cryostatic sections were washed twice in bidistilled water, and then incubated for 10 min in 1% Periodic Acid (Sigma Aldrich, St. Louis, MO, USA). After a rapid wash in bi-distilled water, sections were stained for 20 min with Shiff Solution (Sigma Aldrich, St. Louis, MO, USA) and then washed for 20 min in tap water. Subsequently, slices were dehydrated in increasing ethanol gradients and xilene, and finally mounted with EUKITT. Samples were visualized with a Nikon Eclipse E600 microscope. Images were acquired using a Nikon DMX 1200 digital camera and ACT-1 acquisition software (Nikon, Tokyo, Japan).

### 4.4. MicroRNA Real Time Quantitative RT-PCR

For microRNAs analyses, total RNA was extracted from SVZ and STR tissues (WT, SD and TSD) using the miRCURY RNA isolation KIT (Exiqon-Qiagen, Hulsterweg 82, 5912 PL Venlo, The Netherlands), according to the manufacturer’s protocol and our previous work [85,86,87]. Quality and concentration of purified RNA were evaluated by a BioPhotometer (Eppendorf, Mittelsachsen, Saxony, Germany), using the RNA program. Low molecular weight RNAs were converted to cDNA using miRCURY LNA Universal RT microRNA PCR (Exiqon-Qiagen), following the manufacturer’s protocol. Real-time RT-PCR was performed using specific PCR primer set assay (miRCURY LNA, UniRT microRNA PCR) (Table 3) and relative miRCURY LNA SYBR Green master mix, Universal RT, both from Exiqon, to analyze miRNA expression. The relative quantification of miRNA analyzed in SVZ and striatum region of WT, SD and TSD mice was determined by the comparative 2^−ΔΔCt^ method, where the target is normalized to the reference U6 snRNA, which was determined using the miRCURY LNA U6 snRNA PCR primer set, and relative to WT.

### 4.5. Computational Analysis

#### 4.5.1. miRNA Differential Expression Analysis Profiling

For the analysis of real-time qPCR data, the *Pcr* package (https://CRAN.R-project.org/package=pcr) was used for quality assessing, analyzing and testing data and to identify differential expression of miRNAs between WT and pathological samples. We used the ΔΔ*C*T method and a two-tailed Student’s *t*-test for statistical analysis. The DEMs were considered significantly differentially expressed if the Log2 Fold Change (FC) was >1 or <0.5 and the *p*-value was <0.05.

#### 4.5.2. miRNA-Gene Target Prediction

miRWalk2.0 [88] is a webserver containing novel predicted miRNA-mRNA pairs that are calculated using well-established algorithms, including DIANA-MicroTar, miRanda, miRDB, mirWalk, Pita, RNA22, Targetscan, RNAhybrid among others. For the validated target, three different databases were used: miRecords, miRTarBase, TarBase, which are included in MultiMir R package [89]. Targets gene were defined as genes predicted by at least six algorithms of eight target prediction tools (DIANA-MicroTar, miRanda, miRDB, mirWalk, Pita, RNA22, Targetscan, RNAhybrid) plus all experimentally validated targets.

#### 4.5.3. Gene List Targets Generation and Pathway Enrichment Analysis 

For SVZ-SD, STR-SD, SVZ-TSD, and STR-TSD, we generated a gene list of predicted targets correlated to the specific miRNAs’ expression. We used the DAVID web tool [90] to filter the gene list to predict the gene ontology biological process (GO BP) and to perform pathway enrichment analysis (Panther and Reactome pathways) of all miRNAs that were identified as significantly expressed miRNAs in the differential expression analysis. The gene list was filtered based on predicted targets expressed only in the brain tissue [90].

Further, for testing pathways, GO terms, and for eliminating redundant terms, the PANTHER classification system (v.14.1 released12 March 2019; http://www.pantherdb.org) was used with the test statistics based on gene counts, i.e., Fisher’s exact test *p*-value, with the Benjamini–Hochberg false discovery rate (FDR) correction method [65], based on a previously generated gene list. Only significantly corrected *p*-values (*p*-adjusted < 0.05) and terms annotated to more than 5 and to fewer than 300 genes in our dataset were taken into account.

#### 4.5.4. Gene-miRNA Targets Prediction

To highlight the principal miRNAs and their targets genes involved in a specific pathway, for each enriched pathway, the list of genes was obtained (http://www.pantherdb.org/pathway/; https://www.reactome.org) and a Gene-miRNA target prediction was conducted through mirWalk2.0 [88]. Target miRNAs were defined as miRNA predicted by at least four algorithms of eight target prediction tools (DIANA-MicroTar, miRanda, miRDB, mirWalk, Pita, RNA22, Targetscan, RNAhybrid) plus all experimentally validated targets.

## Figures and Tables

**Table 1:** miRNAs differentially expressed in SVZ and STR areas from SD and TSD mice.

Brain Area	miRNA	Log_2_ FC	*p*-Value
SVZ-SD	miR-9	−1.06	0.027
SVZ-SD	miR-124	1.39	0.005
SVZ-SD	miR-128	1.12	0.044
SVZ-SD	miR-137	−1.69	0.032
STR-SD	miR-33	−1.25	0.043
STR-SD	miR-124	1.03	0.013
STR-SD	miR-126a	−1.12	0.046
SVZ-TSD	miR-29a	1.21	0.038
SVZ-TSD	miR-33	−1.32	0.004
SVZ-TSD	miR-128	−1.16	0.016
STR-TSD	miR-19a	−2.67	0.020
STR-TSD	miR-33	−1.94	0.027
STR-TSD	miR-34a	−2.12	0.030
STR-TSD	miR-124	−2.03	0.005
STR-TSD	miR-128	−1.73	0.017

*p* < 0.05 was considered statistically significant. miRNA, microRNA; FC, fold-change.

**Table 2:** Counts of validated and predicted target genes for each miRNA.

miRNA	Validated Genes	Brain-specific Validated Genes	Predicted Genes	Brain-Specific Predicted Genes	Total Counts/Total Brain Genes
miR-9	531	304	810	370	1341/674
miR-19a	27	17	453	232	480/249
miR-29a	44	6	528	242	572/248
miR-33	16	0	362	164	378/164
miR-34a	56	28	996	477	1052/505
miR-124	461	203	810	360	1271/563
miR-126a	9	0	207	110	216/110
miR-128	56	6	1136	518	1192/524
miR-137	33	19	247	119	280/138

**Table 3:** miScript Primer Assays QIAGEN.

Targets Mature miRNA	Catalogue No.	miRBASE Accession Number	Abbreviation in the Text
mmu-miR-9-5p	MS00012873	MIMAT0000142	miR-9
mmu-miR-9*	MS00005887	MIMAT0000143	miR-9*
mmu-miR-19a-3p	MS00001302	MIMAT0000651	miR-19a
mmu-miR-29a-3p	MS00001372	MIMAT0000535	miR-29a
mmu-miR-33-5p	MS00032697	MIMAT0000667	miR-33
mmu-miR-34a-5p	MS00001428	MIMAT0000542	miR-34a
mmu-miR-124-3p	MS00029211	MIMAT0000134	miR-124
mmu-miR-126a-3p	MS00005999	MIMAT0000138	miR-126a
mmu-miR-128-3p	MS00011116	MIMAT0000140	miR-128
mmu-miR-133a-3p	MS00032305	MIMAT0000145	miR-133a
mmu-miR-137-3p	MS00001589	MIMAT0000149	miR-137
mmu-miR-181c-5p	MS00032382	MIMAT0000674	miR-181c

## Supplementary Materials

Supplementary materials can be found at https://www.mdpi.com/1422-0067/20/13/3179/s1. **Figure S1**. Word cloud based on the literature (open access papers) for each miRNA, and from the searchable database miRBASE (www.mirbase.org). **Figure S2**. Dotplot of enrichment analysis in: (**a**) SD-SVZ compared to SD-STR.; Panther (P) and Reactome (R-MMU) pathways; (**b**) and in TSD-SVZ compared to TSD-STR.; Panther (P) and Reactome (R-MMU) pathways. *p*-adjusted < 0.05 was considered statistically significant. **Table S1**. miRNA-mRNA target-relationship in enriched pathways in SVZ and STR of SD and TSD mice.
