# Supplementary material for: Integrated Computational Analysis Highlights unique miRNA Signatures in the Subventricular Zone and Striatum of GM2 Gangliosidosis Animal Models
Source: Int J Mol Sci. 2019 Jun 28;20(13):3179. doi: 10.3390/ijms20133179 (PMC6651736; doi:10.3390/ijms20133179)
Supplement: Supplementary file 1 [file ijms-20-03179-s001.zip › Morena_et_al_Supplementary_Figures.pdf]

Figure S1

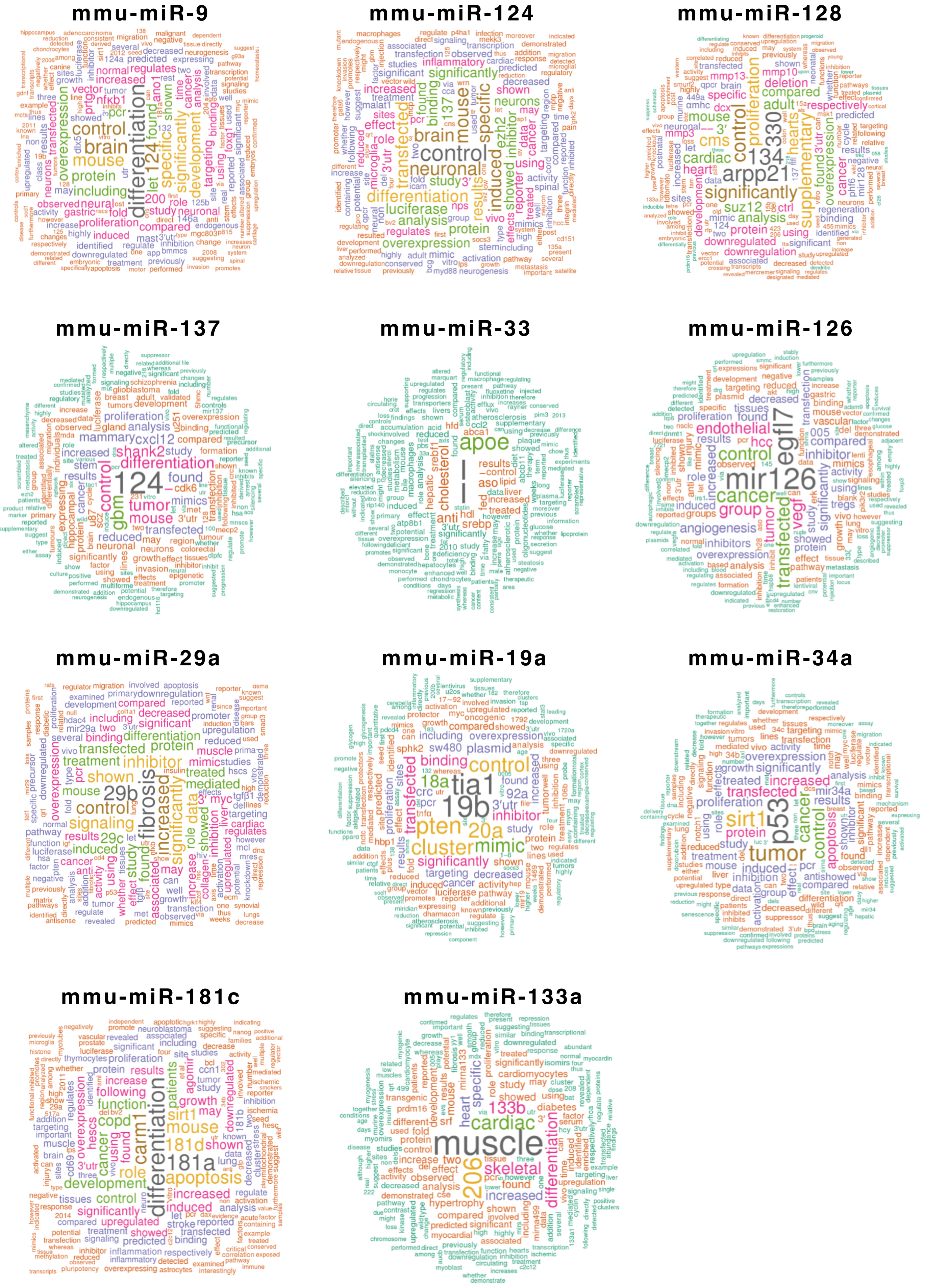

Figure S1. Word cloud based on literature (open access papers) for each miRNA, and from searchable database miRBASE (www.mirbase.org).

Figure S2

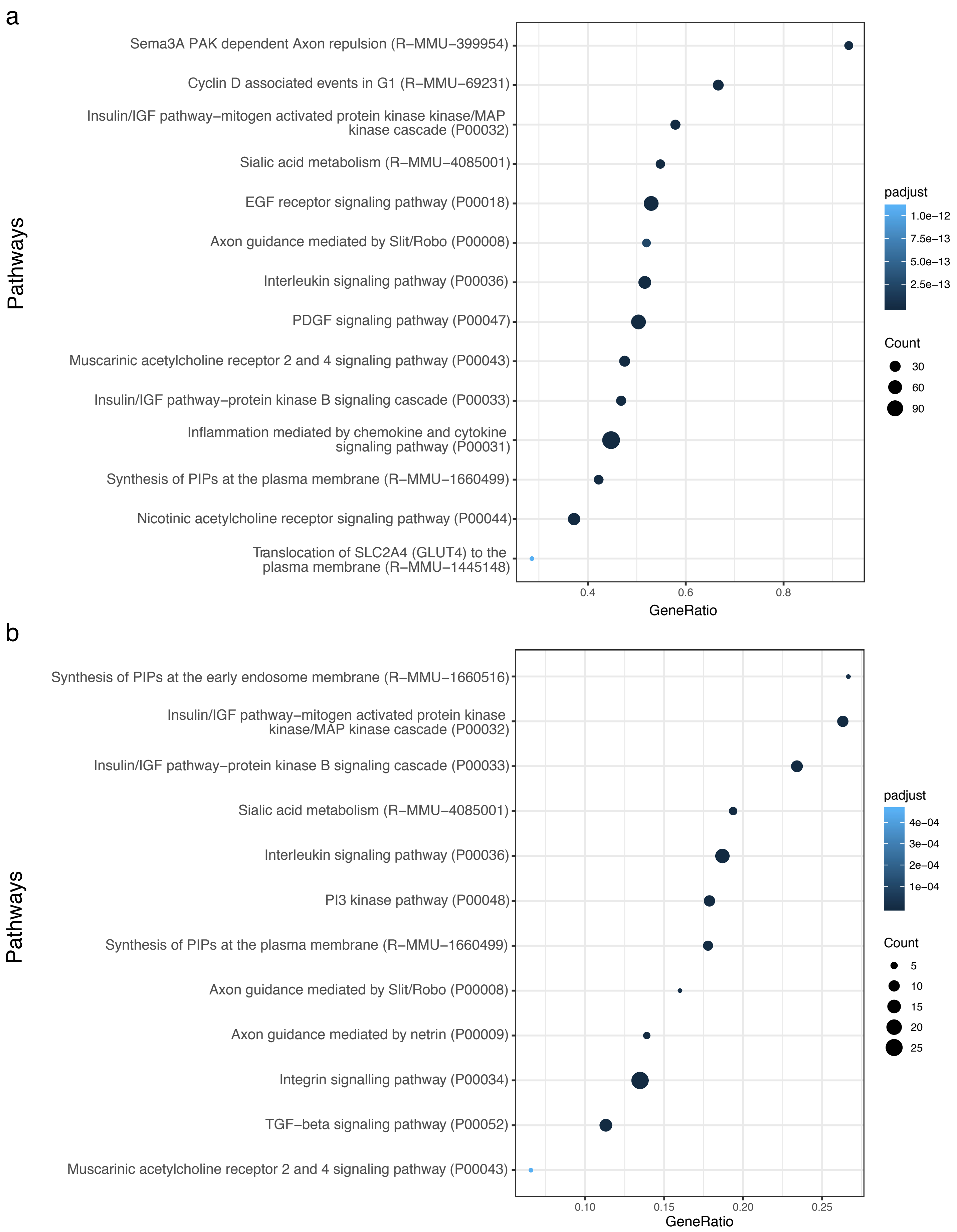

Figure S2. Dotplot of enrichment analysis in: (**a**) SD-SVZ compared to SD-STR.; Panther (P) and Reactome (R-MMU) pathways; (**b**) and in TSD-SVZ compared to TSD-STR.; Panther (P) and Reactome (R-MMU) pathways. Padjusted < 0.05 was considered statistically significant.
